# Sweet Taste Is Complex: Signaling Cascades and Circuits Involved in Sweet Sensation

**DOI:** 10.3389/fnhum.2021.667709

**Published:** 2021-06-22

**Authors:** Elena von Molitor, Katja Riedel, Michael Krohn, Mathias Hafner, Rüdiger Rudolf, Tiziana Cesetti

**Affiliations:** ^1^Institute of Molecular and Cell Biology, Hochschule Mannheim, Mannheim, Germany; ^2^BRAIN AG, Zwingenberg, Germany; ^3^Interdisciplinary Center for Neurosciences, Heidelberg University, Heidelberg, Germany

**Keywords:** sweet taste receptor, signaling, gustducin, calcium, GLP-1, gastro-intestinal tract, brain

## Abstract

Sweetness is the preferred taste of humans and many animals, likely because sugars are a primary source of energy. In many mammals, sweet compounds are sensed in the tongue by the gustatory organ, the taste buds. Here, a group of taste bud cells expresses a canonical sweet taste receptor, whose activation induces Ca^2+^ rise, cell depolarization and ATP release to communicate with afferent gustatory nerves. The discovery of the sweet taste receptor, 20 years ago, was a milestone in the understanding of sweet signal transduction and is described here from a historical perspective. Our review briefly summarizes the major findings of the canonical sweet taste pathway, and then focuses on molecular details, about the related downstream signaling, that are still elusive or have been neglected. In this context, we discuss evidence supporting the existence of an alternative pathway, independent of the sweet taste receptor, to sense sugars and its proposed role in glucose homeostasis. Further, given that sweet taste receptor expression has been reported in many other organs, the physiological role of these extraoral receptors is addressed. Finally, and along these lines, we expand on the multiple direct and indirect effects of sugars on the brain. In summary, the review tries to stimulate a comprehensive understanding of how sweet compounds signal to the brain upon taste bud cells activation, and how this gustatory process is integrated with gastro-intestinal sugar sensing to create a hedonic and metabolic representation of sugars, which finally drives our behavior. Understanding of this is indeed a crucial step in developing new strategies to prevent obesity and associated diseases.

## Introduction

### Increased Sugar Consumption Causes Severe Health Problems

Sugars, as a prime source of calories, are used for metabolic energy production. Perhaps as a consequence of this, sweet taste is one of the most passionate sensations humans experience (DiNicolantonio et al., 2018). Already the human fetus has a preference for sweet compounds present in the amniotic fluid, and neonates show responses to sweet solutions (Tatzer et al., 1985; Steiner et al., 2001) (for review Beauchamp and Mennella, 2011; Ventura and Mennella, 2011). Sugar attraction is generally driven by the activation of brain reward pathways (Araujo et al., 2012; Kendig, 2014; for review Han et al., 2019; Gutierrez et al., 2020) and may lead to addictive behavior (Kendig, 2014). The strong attraction to sugars is partly learned (Veldhuizen et al., 2007) and influenced by many factors, such as other sensory inputs (Ohla et al., 2012) (for review Small, 2012), emotions (Noel and Dando, 2015) and the internal metabolic state (Zhang et al., 2018). Furthermore, also genetics may influence individual variability in sweet taste preference (Keskitalo et al., 2007; Bachmanov et al., 2014).

Only 200 years ago, industrialization and colonial trading increased the global sugar availability by distributing the yield of large sugar cane fields to the world (Tappy, 2012; Chow, 2017). Since then, the consumption of the once luxury product increased steadily. Initially used to sweeten beverages such as tea, coffee and coco, the fabrication of chocolate bars, ice-creams and sodas started in the 20th century (Tappy, 2012). Nowadays, sugar has conquered virtually all food suppliers over the world (DiNicolantonio et al., 2018), and sugar consumption increased from 5 kg/person/year in 1800, to 70 kg/person/year in 2006 (Tappy, 2012). This has contributed to obesity and has become a main risk factor for many chronic disorders including type-2 diabetes, cardiovascular diseases and metabolic syndrome (Bray and Popkin, 2014; Borges et al., 2017; Chow, 2017; Kochem, 2017). However, in contrast to amino acids or fats, which are essential for the body, there is no strict physiological requirement for sugar consumption (Westman, 2002; DiNicolantonio et al., 2018).

To fight the present sugar overload, much effort has been put into finding sugar substitutes, such as non-caloric sweeteners, which are sweet, but contain no calories (Pepino, 2015). Today, there are seven principal non-caloric sweeteners on the market: advantame, saccharin, aspartame, sucralose, cyclamate, neotame and acesulfame K^+^, whose daily acceptable intake dosage is approved by the FDA (Pepino, 2015). However, some of the sweeteners are known for their unpleasant bitter off-taste (Moskowitz and Klarmann, 1975; Kuhn et al., 2004; Galindo-Cuspinera et al., 2006). Although their safety has been clinically assessed (FDA/EFSA) (summarized in BfR Background Information from Bundesinstitut für Riskobewertung, 2014), recent studies suggest that they may increase the risk of cancer, obesity and diabetes. A probable reason for these unexpected side effects might be the activation sweet taste receptors in many extraoral tissues (for review Yamamoto and Ishimaru, 2013; Laffitte et al., 2014). Thus, understanding sweet taste signaling, including its effect in the gastro-intestinal tract and the brain, might help to mitigate the sugar dominance and improve global health.

### Structure of the Taste Buds

According to the current knowledge, sweet taste is first sensed by the taste buds, i.e., gustatory organs, which are formed by roughly 100 specialized taste bud cells each (Lindemann, 1996; Montmayeur, 2002; Roper and Chaudhari, 2017). Humans possess about 5,000 taste buds (Suzuki, 2007; Witt, 2019). Taste bud cells can be grouped into four types (I-IV), defined by their morphology, function and expression profile (for review Roper, 2013): type I cells, with glia-like function; type II (receptor) cells, which stimulate the gustatory nerve terminals via unconventional ATP release upon detection of umami, bitter or sweet stimuli (Finger et al., 2005; Huang et al., 2007); type III cells, which transduce sour taste and make functional synapses with the afferent gustatory nerve fibers (Finger, 2005; DeFazio et al., 2006; Roper, 2013) and finally, type IV basal cells, which serve as progenitor cells (Ren et al., 2014) to replenish mature taste bud cells, as these possess a limited life span (in rodents, half-live varies form 8 to 22 days according to the cell type) (Chaudhari and Roper, 2010; Perea-Martinez et al., 2013; Liman et al., 2014; Barlow, 2015). More recently, a group of broadly responding taste bud cells has been characterized which have a type III phenotype, but respond to multiple gustatory stimuli (Dutta Banik et al., 2020). In mammals, taste buds are located in specialized papillae all over the tongue, epiglottis and palate (Lindemann, 1999; Montmayeur, 2002; Witt, 2019). Fungiform papillae, in the anterior tongue, are innervated by the chorda tympani nerve, a branch of the cranial nerve VII. Circumvallate papillae are located on the dorsal tongue and are in contact with the glossopharyngeal nerve (cranial nerve IX) (Scott, 2005; Kikut-Ligaj and Trzcielińska-Lorych, 2015). Foliate papillae, on the lateral sides of the tongue, are innervated by both nerves (Montmayeur, 2002; Witt, 2019). In the larynx there are taste buds and also single taste cells, which are in contact with the superior laryngeal branch of the vagus nerve (X) (Jowett and Shrestha, 1998; Sbarbati et al., 2004). In addition, sweet compounds stimulate the gastro-intestinal system, the brain, and other organs, either directly or indirectly via gustatory mechanisms (for review von Molitor et al., 2020c). Reciprocal cross talk occurs between oral sweet-sensation and visceral homeostatic signals. Indeed, intestinal hormones and neuropeptides have been identified in taste buds and shown to modulate taste bud cells activity (for review Dotson et al., 2013). In particular, glucagon-like-peptide 1 (GLP-1), leptin and endocannabinoids modulate sweet taste responses (Ninomiya et al., 2002; Shin et al., 2008; Martin et al., 2009; Niki et al., 2010; Martin et al., 2012).

### Studying Taste in Human

Studies on taste transduction in human have progressed slowly for many reasons: (1) taste bud cells make up less than 1% of the tongue, (2) human samples are rare, and (3) primary taste bud cells have a short life span (Liman et al., 2014; Barlow, 2015). Therefore, assessment of human taste physiology has been mostly carried out by *in vivo* “taste sensitivity measurements” which probe the ability of subjects to taste a certain stimulus and determine its quality (Reed and McDaniel, 2006; Aleman et al., 2016). Such tests fall into different categories. In “quality tests” only the taste modality is defined (Galindo-Cuspinera et al., 2006; Zhang et al., 2009). In “detection threshold tests” the lowest concentration of a tastant that a subject can recognize is determined (Reed and McDaniel, 2006; Zhang et al., 2009). In “intensity tests,” participants evaluate the sweetness of molecules by ranking them in a hierarchical order, often relative to a standard (Reed and McDaniel, 2006). Alternatively, sweet taste can be analyzed using “hedonic assessment” (Reed and McDaniel, 2006), where people rate how pleasant a compound is (Kampov-Polevoy et al., 1997) and if it is preferred over another one (Liem and Mennella, 2002; Reed and McDaniel, 2006). Until now, assays to understand the underlying intracellular signaling and/or neuronal pathways are very difficult to pursue in humans. However, the sweet taste receptor inhibitor lactisol has been used in humans to investigate the perception of polysaccharides (Lapis et al., 2016; Schweiger et al., 2020). Further, a blue food-dye (Robert’s Brilliant Blue FCF133) can be used for live staining of tongue papillae in humans (Shahbake et al., 2005; Zhang et al., 2009; Gardner and Carpenter, 2019). In addition, with brain imaging techniques, such as MRI (magnetic resonance imaging) and PET (positron emission tomography), the brain regions activated by sweet stimuli have been mapped in humans (Prinster et al., 2017; Canna et al., 2019; Avery et al., 2020) (for review Han et al., 2019).

Due to these limitations, taste-related signaling mechanisms have been studied mainly in rodents, although there are major species-related differences. For example, rodents have a much stronger preference for polysaccharides compared to humans (Feigin et al., 1987). Further, certain sweet taste receptor inhibitors are species specific, such as gurmarin for rodents and lactisol for humans (Hellekant, 1976; Hellekant et al., 1988; Jiang et al., 2005). An alternative experimental system consists in mammalian cell lines heterologously expressing the human sweet taste receptor and its downstream signaling molecules. In this case however, the native cellular background and the niche are missing (von Molitor et al., 2020b). Thus, a new approach, based on organoids derived from mouse taste progenitor cells, may resemble more closely the native environment (Ren et al., 2009, 2010, 2014, 2017) and organoids could be theoretically also generated from human papillae. Another recent approach consists in the generation of a stably proliferating cell line from human lingual cells, that can be used to produce 3D-cell cultures, such as spheroids (Hochheimer et al., 2014; von Molitor et al., 2020a). Thus, an optimal model to study sweet taste transduction, especially in human, has still to be established.

## A Long Way to the Discovery of the Sweet Taste Receptor

Long before the major components of taste transduction pathways were unraveled, Hänig showed that different tongue areas were more sensitive to certain taste modalities (Hanig, 1901). Unfortunately, many years later his experimental line-graph was redrawn in a simplified and mispresenting manner (Boring, 1942), leading to the common and long-lasting erroneous belief that the five taste modalities (sweet, bitter, umami, sour, salt) map to distinct tongue areas (Schiffman et al., 1986; Hoon et al., 1999). Finally, in 1974, evidence was provided that each taste modality can be sensed on every tongue part, but with different detection thresholds (Collings, 1974). Regarding sweetness, the nerve with the highest sensitivity is the glossopharyngeal in rats (Krimm et al., 1987), and chorda tympani in mice and rhesus monkeys (Hellekant et al., 1997; Danilova and Hellekant, 2003). In humans, since nerve recordings are not possible, contrasting results were obtained: the sweet detection threshold was reported to be lower either at the posterior tongue (Dastur, 1961; Okuda and Tomita, 1976) or at the tongue tip (Collings, 1974), while others reported no spatial difference (Nilsson, 1979; Sato et al., 2002). Nonetheless, subregional differences were detected even within the anterior tongue, with the edge and the lateral regions being the most sweet-sensitive areas (Stein et al., 1994). A reason for the divergence could be the heterogeneity of subjects, since taste is influenced by age (Stein et al., 1994; Ng et al., 2004), genetic variance (Bachmanov et al., 2014; Eriksson et al., 2019; Hwang et al., 2019), sex (Than et al., 1994; Fushan et al., 2010), diseases (Ng et al., 2004), and temperature (Talavera et al., 2005; Lemon, 2015).

Before the sweet taste receptor was discovered, sweet transduction in taste bud cells was proposed to involve cyclic adenosine monophosphate (cAMP) and protein-kinase A (PKA). In general, cAMP levels are regulated by its synthesis via adenyl cyclases (ACs) and hydrolysis via phosphodiesterases (PDEs) (Trubey et al., 2006). Already in 1972, a high AC activity was found in bovine tongue epithelium, enriched in taste buds (Kurihara and Koyama, 1972). Consistently, rat taste bud cells express AC 4,5 and 8 (Abaffy et al., 2003; Trubey et al., 2006). Sugars and saccharin were shown to stimulate AC in the presence of guanine nucleotides in frog, rat and pig tongue epithelium (Avenet and Lindemann, 1987; Striem et al., 1989; Naim et al., 1991; Striem et al., 1991). Electrophysiology studies revealed that application of cAMP analogs caused taste bud cell depolarization due to reduced K^+^ outward currents via PKA-dependent phosphorylation (Avenet and Lindemann, 1987; Tonosaki and Funakoshi, 1988; Striem et al., 1991). Consistently, in rat and hamster taste bud cells, saccharin and sucrose elicited depolarization and generated action potentials, an effect that was mimicked by the application of a permeable analog of cAMP and cGMP and did not require extracellular Ca^2+^ (Béhé et al., 1990; Cummings et al., 1993). A different mechanism was unraveled in frog taste bud cells where saccharin and NC01 stimulation resulted in PDE-mediated cAMP hydrolysis, which in turn activated a cyclic-nucleotide-suppressible channel (CNG), mediating Ca^2+^ influx and cell depolarization (Kolesnikov and Margolskee, 1995). A direct proof of sweet-mediated cAMP/PKA pathway activation in taste bud cells is therefore still missing, hampered at that time by technical limitations, and later on possibly neglected.

In 2001, a big breakthrough was finally achieved, when multiple groups identified the “sweet taste receptor”: a heterodimer formed by two G-protein coupled receptor (GPCR) subunits, T1R2 and T1R3, located at the taste pore of type II taste bud cells (Hoon et al., 1999; Bachmanov et al., 2001; Kitagawa et al., 2001; Max et al., 2001; Montmayeur et al., 2001; Nelson et al., 2001; Li et al., 2002) (for review Temussi, 2006; DuBois, 2016). This discovery was based on the observation that two mouse strains, called tasters (C57BL/6 and DBA/2), were strongly attracted by saccharin and D-phenylalanine (Bachmanov et al., 1997). *Sac* and *dpa*, which are both located on chromosome 4, were identified as the main loci determining sweet preference in mice (Fuller, 1974; Capeless and Whitney, 1995; Lush et al., 1995; Max et al., 2001; Margolskee, 2002; Shigemura et al., 2005). Consistently, both genes were found to influence peripheral nerve response to sucrose (Bachmanov et al., 1997). The first sweet-related subunit cloned was T1R2, however, its function was not clear at that time (Hoon et al., 1999). Soon afterward, *Tas1r3*, the gene encoding the T1R3 subunit, was mapped on the human chromosome 1p36, and based on this sequence the murine ortholog was found in the *Sac* locus (Fuller, 1974; Lush, 1989; Lush et al., 1995; Kitagawa et al., 2001; Montmayeur et al., 2001; Nelson et al., 2001; Sainz et al., 2001). Commonly, heterodimers of T1R2 and T1R3 form functional sweet taste receptors, as demonstrated with recombinant systems (Nelson et al., 2001; Li et al., 2002; Zhao et al., 2003) and transgenic mouse models (Zhao et al., 2003). The T1R family contains one additional subunit: T1R1, that forms with T1R3 the umami receptor (Nelson et al., 2001; Zhao et al., 2003). All three T1R subunits belong to the class C GPCRs. Immunostainings and *in situ* hybridization in mouse tongue revealed the expression of T1R3 in about one third of taste bud cells in almost all papillae (Table 1; Nelson et al., 2001). Even if bitter- (T2R) and sweet taste receptors (T1R2/T1R3) were both present in type II cells, their expression did not overlap (Nelson et al., 2001). Furthermore, also sweet (T1R2) and umami (T1R1) specific subunits were mainly present in distinct type II cell populations (Hoon et al., 1999; Stone et al., 2007). Interestingly, fitting the old observations of Hänig, regional differences in the expression were recognized (Table 1). In rodents, T1R3 was present in all papillae, but the strongest expression was observed in circumvallate and foliate papillae, where also T1R2 showed the highest and almost exclusive expression (Montmayeur et al., 2001; Nelson et al., 2001) (for review Montmayeur, 2002). In human, T1R3 was detected in circumvallate and fungiform papillae (Max et al., 2001). Thus, the heterodimeric T1R2/T1R3 GPCR was recognized as the main molecular sensor for sugars and other sweet compounds.

**TABLE 1:** Expression of the sweet taste receptor subunits (T1R2/T1R3) in mammal taste papillae.

*Subunit*	*CV*	*Fungiform*	*Foliate*	*Palatal*	*Species*	*Source*
*T1R3*	strong	strong	strong		Mouse	Kitagawa et al., 2001
	∼30% cells	∼30% cells	∼30% cells	∼30% cells	Mouse	Nelson et al., 2001
	24% cells	15% cells	14% cells		Mouse	Montmayeur et al., 2001
	strong	strong	strong		Mouse	Max et al., 2001
	20% cells	20% cells			Human	Max et al., 2001
	100% TB, 23% cells	<4% TB, <1% cells	100% TB, 26% cells		Mouse	Sainz et al., 2001
	strong	no	Less strong	No	Mouse	Matsunami et al., 2000
	6288 × 10^–7^	150600 × 10^–7^	30 × 10^–7^		Mouse	Choi et al., 2016
*T1R2*	all TBs 20–30% cells	0.5% cells	abundant	few	Rat	Hoon et al., 1999
	yes	yes	yes		Mouse	Nelson et al., 2001
	yes	no	yes		Mouse	Kitagawa et al., 2001
	strong	low	strong		Mouse	Montmayeur et al., 2001
	strong	less strong	few	few	Mouse	Matsunami et al., 2000
	7180 × 10^–7^	21 × 10^–7^	1170 × 10^–7^		Mouse	Choi et al., 2016

*Percentage refers to the cells or to the taste bud (TB), as reported. CV, circumvallate papillae. The expression was detected by in situ hybridization (Hoon et al., 1999; Kitagawa et al., 2001; Max et al., 2001; Montmayeur et al., 2001; Nelson et al., 2001; Sainz et al., 2001), immunostaining (Max et al., 2001), PCR (Matsunami et al., 2000), and qPCR (Choi et al., 2016).*

## Sweet Taste Receptor Mediated Transduction

Not only natural sugars, such as monosaccarides or disaccharides, activate the sweet taste receptor, but also ligands with very different chemical structures, such as amino acids, proteins and non-caloric sweeteners (McCaughey, 2008), may bind to different domains of T1R2/T1R3 (Sainz et al., 2007; DuBois, 2016). In particular, in transgenic cells and animals it was shown that the human T1R2 (hT1R2) confers sensitivity to aspartame, glycyrrhizic acid, monellin and thaumatin (Zhao et al., 2003), while hT1R3 contains a binding site for neohesperidin dihydrochalcone (Li et al., 2002). It is not yet clear if also oligosaccharides, such as starch, are able to activate the sweet taste receptor (Lapis et al., 2016; Schweiger et al., 2020). In general, the change in the sweet taste receptor conformation upon ligand binding activates an intracellular downstream signaling.

### The Canonical Signaling Pathway for Sweet and Bitter Taste

**FIGURE 1:**
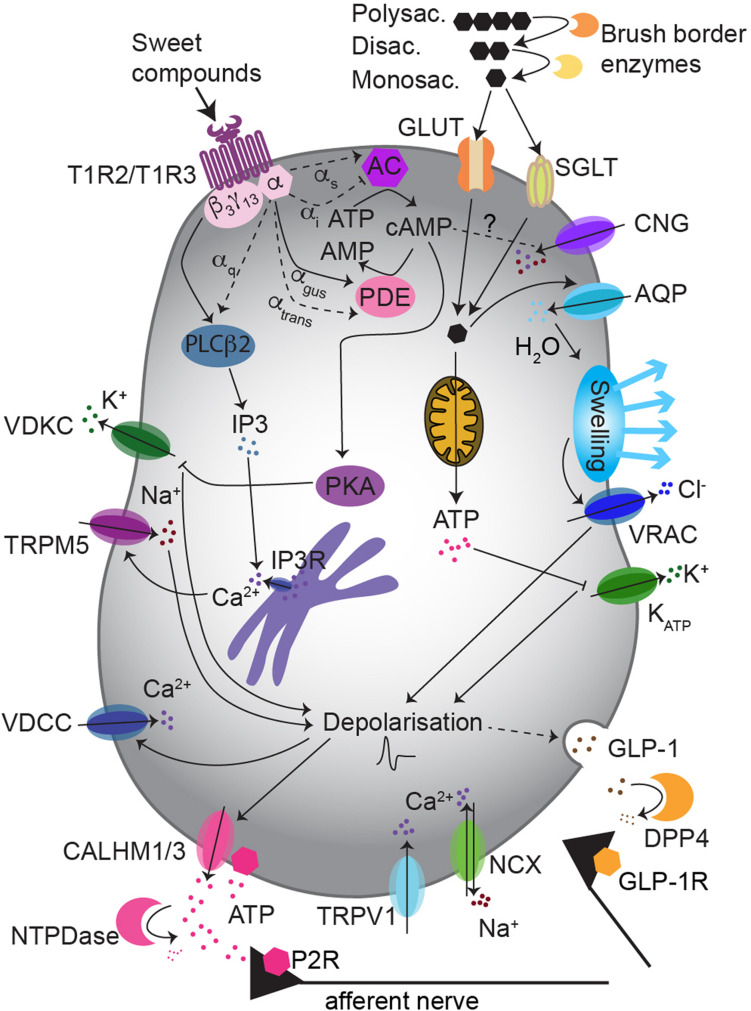
Sweet taste transduction uses multiple pathways in type II taste bud cells. The “canonical pathway” implies the activation of gustducin by T1R3/T1R2 receptor, which then promotes: intracellular Ca^2+^ rise via PLCβ2/IP3 signaling, cell depolarization via TRPM5 and ATP release. Non-caloric sweeteners may preferentially use the PLCβ2/IP3 pathway, while sugars may rather activate a cAMP/PKA pathway, depolarizing the cell via K^+^ channels inhibition. The “alternative pathway” possibly involves glucose influx via GLUTs and/or SGLT1, increase of ATP and inhibition of K_*ATP*_-mediated K^+^ outflow. This may induce GLP-1 release. Abbreviations: AQP, aquaporins; DPP4, dipeptidyl peptidase 4; CALHM1/3, Ca^2+^ homeostasis modulator 1/3; VDKC, voltage-dependent K^+^ channel; P2R, purinergic receptor class 2; GLP-1R, GLP-1 receptor.

Sweet and bitter transduction pathways have been discovered in parallel and they share many components. In human, bitter compounds bind to a variety of ∼25 different receptors of the T2R family, that can form both homomeric and heteromeric complexes (Kuhn et al., 2004; Kuhn and Meyerhof, 2013). T2R activation leads to the release of the β_3_γ_13_ subunit (Huang et al., 1999; Rössler et al., 2000) from the associated G-protein, which then activates phospholipase Cβ2 (PLCβ2) (Rössler et al., 1998; Miyoshi et al., 2001; Yan et al., 2001; Zhang et al., 2003) to generate inositol-3-phosphate (IP3) (Rössler et al., 1998; Miyoshi et al., 2001; Yan et al., 2001; Zhang et al., 2003). Subsequently, IP3 binds to its receptor (IP3R) on the endoplasmic reticulum (Clapp et al., 2001; Miyoshi et al., 2001) and induces Ca^2+^ release from the stores (Akabas et al., 1988). Increased cytoplasmic Ca^2+^ levels in turn open the membrane-associated transient receptor potential channel (TRPM5) (Pérez et al., 2002; Zhang et al., 2003) permitting Na^+^ influx, followed by cell depolarization and ATP release via CALHM1/3 channel (Taruno et al., 2013; Table 2 and Figure 1). The signaling network downstream the sweet taste receptor involves the same key players (Table 2) and it is known as the “canonical pathway.” The functional role of these signaling molecules has been tested in several knockout mouse models, which displayed abolished or reduced nerve and behavioral responses to sweet compounds (Table 2) (for review von Molitor et al., 2020c).

**TABLE 2:** Overview of signaling molecules involved in bitter and sweet signaling.

*Signaling molecule*	*Bitter*	*Sweet*
*Gβ_3_*	Rössler et al., 2000	Max et al., 2001
*Gγ_13_*	Huang et al., 1999	Max et al., 2001
*PLCβ2*	Rössler et al., 1998; Miyoshi et al., 2001; Yan et al., 2001; Zhang et al., 2003	Asano-Miyoshi et al., 2000; Max et al., 2001; Miyoshi et al., 2001; Zhang et al., 2003
*IP3*	Hwang et al., 1990; Ogura et al., 1997; Spielman, 1998; Huang et al., 1999	Bernhardt et al., 1996; Uchida and Sato, 1997; Usui-Aoki et al., 2005
*IP3R*	Clapp et al., 2001; Miyoshi et al., 2001	Miyoshi et al., 2001
*Ca^2+^ release from stores*	Akabas et al., 1988	Bernhardt et al., 1996; Uchida and Sato, 1997
*TRPM5*	Pérez et al., 2002; Zhang et al., 2003	Pérez et al., 2002; Zhang et al., 2003; Talavera et al., 2005

*Involvement of the signaling molecules was demonstrated with knockout mouse models, where Ca^2+^ signals in taste bud cells, nerve responses and/or behavioral attraction were measured upon stimulation. Immunostainings were used for localization of the signaling molecules.*

### Sweet Transduction May Involve Multiple G-proteins

The first described G-protein coupled to the sweet taste receptor was gustducin: a G-protein related to the G_*i*_ family, which consists of Gα-gustducin (Gα_*gust*_) and Gβ_3_γ_13_ (McLaughlin et al., 1992; Huang et al., 1999). Gustducin is specifically expressed in the taste papillae and is closely related to the retinal transducin (McLaughlin et al., 1992; Margolskee, 1993). Gustducin and transducin share 80% sequence identity and many features, such as interaction with βγ subunits, GTPase activity and PDE activation (McLaughlin et al., 1993; Hoon et al., 1995; Ruiz-Avila et al., 1995). A taste specific PDE was found in bovine and rat taste tissue, and gustducin was shown to be interchangeable with transducin in a recombinant baculovirus system (Law and Henkin, 1982; McLaughlin et al., 1994; Ruiz-Avila et al., 1995). In taste buds, the expression ratio transducine/gustducin is 1/25 (McLaughlin et al., 1993; Hoon et al., 1995; Ruiz-Avila et al., 1995). The strongest evidence that the sweet taste receptor can functionally couple to Gα_*gust*_ comes from experiments in recombinant systems, where T1R2/T1R3 was coexpressed with Gα_15_ or the artificial chimeric Gα_16_ subunit derived from murine hematopoietic cells (Nelson et al., 2001, 2002). Still, the functional coupling of Gα_*gust*_ and the sweet taste receptor in native taste tissue is an open issue, as sucrose and non-caloric sweeteners were unable to activate gustducin in bovine taste membrane extract (Ruiz-Avila et al., 1995; Ming et al., 1998).

Further observations suggested that gustducin may be one, but not the only player in sweet taste transduction, since: (1) only a subset of sweet taste receptor expressing cells are positive for gustducin, with publications reporting from 1/10 to 2/3 of double positive cells (Hoon et al., 1999; Montmayeur et al., 2001; Max et al., 2001), and (2) in gustducin-knockout mice the responses to sweet compounds were reduced but not abolished (Wong et al., 1996; Ruiz-Avila et al., 2001; He et al., 2002; Danilova et al., 2006). Regarding Gα_*gust*_ expression, regional differences occur and they are species-specific. In rats, taste buds of the fungiform papillae contain three time less gustducin-positive cells than those of the circumvallate papillae and the palate (Boughter et al., 1997). By contrast, in mice, Gα_*gust*_ is coexpressed with T1R2/T1R3 in fungiform papillae and palatal taste bud cells, but not in the circumvallate papillae (Kim et al., 2003; Stone et al., 2007). This is consistent with the observation that in gustducin-knockout mice electrophysiological recordings from the chorda tympani nerve, showed an almost abolished response to sweet compounds (Wong et al., 1996), while the response of the glossopharyngeal nerve was less affected (Danilova et al., 2006). This suggests that sweet taste transduction may use different pathways according to the location of the taste bud cells, with species-specific differences. Little can be said about gustducin’s functional role in humans as there is only one immunostaining study showing its expression in circumvallate and foliate papillae (Takami et al., 1994).

Gustducin is activated when an agonist binds to a bitter-, sweet- or umami-taste receptor. The conformational change of the GPCR induces GDP/GTP exchange on the Gα subunit, which then dissociates to transduce the signal into the cell (Hoon et al., 1995). For bitter stimuli, the Gα and βγ subunits were proposed to activate distinct downstream effector molecules (Sainz et al., 2007): Gα may induce PDE-mediated cAMP hydrolysis, while βγ may activate PLCβ2/IP3 signaling (Yan et al., 2001). Further, gustducin-knockout mice had elevated basal cAMP levels and bitter responses were unmasked only upon inhibition of PKA (Clapp et al., 2008), proposing that Gα_*gust*_ activates PDEs and is important to maintain low levels of cAMP in resting states (Lindemann, 1996; Spielman, 1998; Clapp et al., 2008). Unfortunately, this scenario was not investigated for sweet taste responses therefore, we can only speculate whether the sweet-mediated pathway similarly requires gustducin to expand the functional range of cAMP changes. Nevertheless, this hypothesis provides a framework for understanding the reduced sweet preference in gustducin-knockout mice (Wong et al., 1996).

The picture is further complicated by the fact that additional G-proteins have been found in taste bud cells (Table 3). Besides Gα_*gust*_, also the mRNA for Gα_*i–*__2_, Gα_*i–*__3_, Gα_*s*_ and Gα_14_ was found in rat taste tissue (McLaughlin et al., 1992). Immunostaining analysis showed that also Gα_15_ and Gα_*q*_ were localized in rat taste buds (Kusakabe et al., 1998). The expression of Gα_*i–*__2_, Gα_*i–*__3_, Gα_*s*_, and Gα_*gust*_ was confirmed in rat circumvallate papillae with different techniques, proving that one taste bud cell can coexpress multiple Gα subunits, and Gα_*gust*_ may not be the dominant species (Kusakabe et al., 1998; Kusakabe et al., 2000). This finding suggests that different, mutually interacting pathways might coexist in individual taste bud cells. Conversely, other evidence supports the idea that multiple pathways may be segregated in different cell subpopulations: for example in mouse taste bud cells, Gα_14_ was found to be coexpressed with T1R3, but not with gustducin (Shindo et al., 2008; Tizzano et al., 2008). The Gα_14_ subunit may be involved in PLC activation and IP3 generation (Kusakabe et al., 1998; Shindo et al., 2008; Tizzano et al., 2008). Based on a differential hybridization screen, Gβ_3_γ_13_ expression was detected in all Gα_*gust*_ positive receptor cells (Rebecchi and Pentyala, 2000). Additionally, also the Gβ_1_γ_13_ subunit was found in receptor cells, and stimulation with a bitter compound revealed its functionality (Huang et al., 1999). Gγ_13_ colocalizes with Gα_*gust*_ and mediates production of IP3 via PLCβ2 upon bitter stimulation (Yan et al., 2001). Finally, also Gγ_3_ was found in taste bud cells, coexpressed with PLCβ2, Gα_*gust*_ and Gβ_3_ (Rössler et al., 2000). Hence, this plethora of observations suggests a heterogenous picture, with several G-proteins and multiple downstream signaling options involved in the sweet taste pathway.

**TABLE 3:** Overview of G-protein subunits expression in the taste buds.

*Trimeric G proteins*	*Species*	*Source*
*Gα_*gust*_*	Recombinant in baculovirus	Hoon et al., 1995
	Mouse	Wong et al., 1996; Huang et al., 1999; Ruiz-Avila et al., 2001; He et al., 2002; Kim et al., 2003; Danilova et al., 2006; Miura et al., 2007; Stone et al., 2007
	Rat	McLaughlin et al., 1992, 1994; Yang et al., 1999; Kusakabe et al., 2000
	Human	Max et al., 2001
*Gα_*trans*_*	Mouse	He et al., 2002
	Rat	Ruiz-Avila et al., 1995; Yang et al., 1999
	Bovine	Ruiz-Avila et al., 1995
*Gα_*s*_*	Rat	McLaughlin et al., 1992, 1994; Kusakabe et al., 2000
*Gα_*i–2*_*	Rat	McLaughlin et al., 1992, 1994; Asano-Miyoshi et al., 2000; Kusakabe et al., 2000
*Gα_*i–3*_*	Rat	McLaughlin et al., 1992, 1994; Kusakabe et al., 2000
*Gα_*i–4*_*	Rat	McLaughlin et al., 1992, 1994
*Gα_12_*	Rat	McLaughlin et al., 1992, 1994
*Gα_15_*	Mouse	Shindo et al., 2008; Tizzano et al., 2008
*Gα_*q*_*	Rat	Kusakabe et al., 1998
*β_3_γ_13_*	Mouse, human	Huang et al., 1999
*β_3_γ_13_*	Rat	Rössler et al., 2000

*The expression was detected by immunofluorescence (Ruiz-Avila et al., 1995; Kusakabe et al., 2000; Max et al., 2001; Shindo et al., 2008), in situ hybridization (Yang et al., 1999; Kusakabe et al., 2000; Kim et al., 2003; Shindo et al., 2008), PCR (Kusakabe et al., 1998; Tizzano et al., 2008), Southern (Kusakabe et al., 2000), and Northern blot (Kusakabe et al., 1998), as well as gene expression profiling (Huang et al., 1999).*

In summary, it has been generally accepted that sweet taste transduction in taste bud cells is mediated by Gα_*gust*_. However, there are still many open questions, and several factors have to be considered in the interpretation of this transduction model: (1) functional studies are still very limited and mainly conducted in recombinant systems, (2) gustducin-knockout mice showed residual sweet taste responses, (3) besides Gα_*gust*_, also other Gα_*q*_ and Gα_*i*_ subunits have been found in taste bud cells (Table 3), (4) several second messengers are mobilized by sweet taste receptor activation. One could speculate that the relative importance of a certain signaling pathway varies among species. Indeed, in mice the Gβγ pathway may dominate, while in rats and hamsters the Gα pathway seems to be more prominent (Trubey et al., 2006). Therefore, it is likely that several G-proteins and different pathways are involved in sweet taste transduction, which may be similar for human sweet taste sensation as well.

## The Sweet Taste Receptor-Independent Pathway

### Residual Sugar Attraction in Sweet Taste Receptor Deficient Mice

After the discovery of the sweet taste receptor, it has been assumed that the taste of sugars and of non-caloric sweeteners is almost exclusively transduced via the heterodimer T1R2/T1R3. However, additional mechanisms mediating sugar perception in taste bud cells have been reported. Indeed, a small residual response to highly concentrated sugars, but not to non-caloric sweeteners, was observed in T1R2 and T1R3 single-knockout mice (Zhao et al., 2003). In another T1R3-knockout strain, no response to non-caloric sweeteners was observed, but chorda tympani responses to disaccharides were only moderately diminished and those to glucose were even preserved (Damak et al., 2003). Further evidence in favor of a sweet-sensing pathway independent of the classical sweet taste receptor, comes from knockout mice models for crucial downstream signaling molecules, such as PLCβ2 (Zhang et al., 2003; Dotson et al., 2005), TRPM5 (Zhang et al., 2003; Talavera et al., 2005; Damak et al., 2006; Sclafani et al., 2007; Ren et al., 2010; Eddy et al., 2012) or gustducin (Wong et al., 1996; He et al., 2002; Ruiz et al., 2003; Glendinning et al., 2005; Danilova et al., 2006; Sclafani et al., 2007), in which behavioral and nerve responses to sugars were not completely abolished (for review von Molitor et al., 2020c). Thus, it was proposed that, although the canonical T1R2/T1R3-mediated pathway is of principal importance for sweet sensation, additional sweet taste receptor independent pathways might sense caloric sugars (von Molitor et al., 2020c). These might employ T1R3 homodimers and/or completely different downstream signalings.

### The Sweet Taste Receptor Independent Pathway May Use Glucose Transporters

In search of potential candidates for such alternative pathways, tissues involved in glucose homeostasis can be taken as models. Metabolic homeostasis in the body is achieved upon glucose absorption in the gastro-intestinal tract and glycemia regulation via pancreatic insulin release. Accordingly, gastro-intestinal and pancreatic cells use specialized mechanisms to sense and take up glucose. These include glucose transporters (GLUTs) and sodium-driven glucose symporters (SGLTs). The GLUT family contains 13 members with tissue-specific expression and functional diversity (Table 4). The SGLTs comprise only three family members (Scheepers et al., 2004; Zhao and Keating, 2007; Deng and Yan, 2016). In β-cells, GLUT2-mediated glucose entry elevates, via oxidative metabolism, the intracellular ATP level, which in turn leads to K_*ATP*_ channel inhibition (Ashcroft et al., 1984; Miki et al., 1998). This drives cell depolarization and triggers insulin release (Ashcroft, 2005; Yamamoto and Ishimaru, 2013; Laffitte et al., 2014). Alternatively, at hyperglycemic conditions, β-cell depolarization occurs via osmotic swelling and consequent activation of volume-regulated anion channels (VRACs), that mediates depolarizing outward Cl^–^ currents (Matsumura et al., 2007; Best et al., 2010; Louchami et al., 2012). Conversely, SGLT1 activation directly depolarizes the cells since glucose entry is coupled to the influx of Na^+^. Furthermore, in contrast to GLUT, SGLT1 is activated also by non-metabolizable glucose analogs (Sclafani et al., 2020; Yasumatsu et al., 2020). In general, GLUTs and SGLT1 shuttle caloric sugars with different affinities (Table 3), but not non-caloric sweeteners. Considering the similarities between the taste papillae and the epithelia of the gastro-intestinal system (see chapter 7), the possibility that glucose transporters may be responsible for the residual response to sugars, as observed in sweet taste receptor deficient mice, was explored.

**TABLE 4:** GLUTs and SGLT expression in taste bud cells.

*Transporter*	*Substrate*	*Species*	*Papillae*	*Source*
*GLUT2 (Slc2A2)*	glucose, mannose, galactose, fructose, glucosamine (Thorens, 2015)	Mouse	CV, foliate, fungiforme	Yee et al., 2011
		Rat	CV	Merigo et al., 2011
*GLUT4 (Slc2A4)*	glucose, dehydroacetic acid (Huang and Czech, 2007; Vargas et al., 2019)	Mouse	CV, foliate, fungiform	Yee et al., 2011
*Glut5 (Slc2A5)*	fructose (Douard and Ferraris, 2008)	Rat	CV	Merigo et al., 2011
*Glut8 (Slc2A8)*	glucose, fructose, galactose (Schmidt et al., 2009)	Mouse	CV, foliate, fungiform	Yee et al., 2011
		Macaque	CV, fungiform	Hevezi et al., 2009
*Glut9 (Slc2A9)*	glucose, fructose, urate (Doblado and Moley, 2009)	Mouse	CV, foliate, fungiform papillae	Yee et al., 2011
*Glut10 (Slc2A10)*	glucose, galactose (Dawson et al., 2001)	Macaque	CV, fungiform	Hevezi et al., 2009
*Glut13 (Slc2A13)*	glucose, IP3 (Zhao and Keating, 2007)	Macaque	CV, fungiform	Hevezi et al., 2009
*SGLT1 (Slc5)*	glucose, galactose (Sabino-Silva et al., 2010; Wright et al., 2011)	Mouse	CV, foliate, fungiform	Yee et al., 2011
		Rat	CV papillae	Merigo et al., 2011

*Expression was detected by PCR (Merigo et al., 2011; Yee et al., 2011), immunohistochemistry (Merigo et al., 2011; Yee et al., 2011), or an affymetrix genome wide array (Hevezi et al., 2009). CV, circumvallate papillae.*

Notably, in human taste bud cells, glucose absorption has been long known (Kurosaki et al., 1998; Oyama et al., 1999; Toyono et al., 2011; Yee et al., 2011) and expression of both SGLT1 and GLUTs, was observed in taste papillae of different species (Table 4). Fittingly, also potential downstream players of GLUTs were found in taste bud cells (for review von Molitor et al., 2020c), supporting the hypothesis that GLUT and SGLT1 may be responsible for the residual glucose preference in T1R3-knockout mice (Damak et al., 2003). Specifically, the involvement of SGLT1 in T1R3-independent sugar responses in mice was recently reported. Yasumatzu et al. showed that NaCl selectively increased sweet responses to glucose and sucrose, but not to non-caloric sweeteners, nor to other taste modalities, both in wild type and T1R3-knockout mice (Yasumatsu et al., 2020). This increase was ablated by phlorizin, a SGLT1 blocker. Additionally, afferent sweet-responsive fibers showed three different response patterns to sweet stimuli: (1) fibers with a maximal response to sugars were sensitive to NaCl and phlorizin, (2) fibers with a maximal response to non-caloric sweeteners were unaffected by NaCl and phlorizin, and (3) fibers with a mixed behavior also responded to NaCl and phlorizin (Yasumatsu et al., 2020). This suggests that there are some sweet-responding taste bud cells expressing only SGLT1, some that express only T1R2/T1R3 and a third group expressing both (Yasumatsu et al., 2020). Whether SGLT1-expressing cells represent type II or another cell population, such as the recently discovered “broadly responsive” cells, needs further investigation (Dutta Banik et al., 2020; Yasumatsu et al., 2020). It is still unknown if the SGLT1-mediated pathway induces Ca^2+^ signals, TRMP5 activation and ATP release, or uses a completely different intracellular mechanism and neurotransmitter. In further support of the SGLT1 hypothesis, all sweet-responsive afferent fibers in T1R3-knockout mice were phlorizin sensitive and could be activated by the non-metabolizable sugar αMDG. In addition, NaCl increased licking of glucose solution in T1R3-knockout animals, an effect blocked by phlorizin (Yasumatsu et al., 2020), providing evidence that the SGLT1-based pathway mediates sugar attraction. Theoretically, also disaccharides could be detected via the alternative pathway. Indeed, mouse taste cells express a group of disaccharidases, called “brush border” enzymes, that hydrolyze the disaccharides to monosaccharides, which in turn can enter the taste cells via the transporters (Merigo et al., 2009; Sukumaran et al., 2016). Additionally, live-cell imaging in taste bud cells using a FRET-based glucose sensor (Deuschle et al., 2005) or (NAD(P)H,FAD) fluorescent imaging, may help to understand the contribution of SGLT1 and GLUTs to sweet responses. To unravel whether the alternative pathway also generates Ca^2+^ events to transduce gustatory responses, live Ca^2+^ imaging should be performed in taste bud cells of T1R3-knockout mice.

### Neurotransmitter and Physiological Role of the Alternative Pathway

**FIGURE 2:**
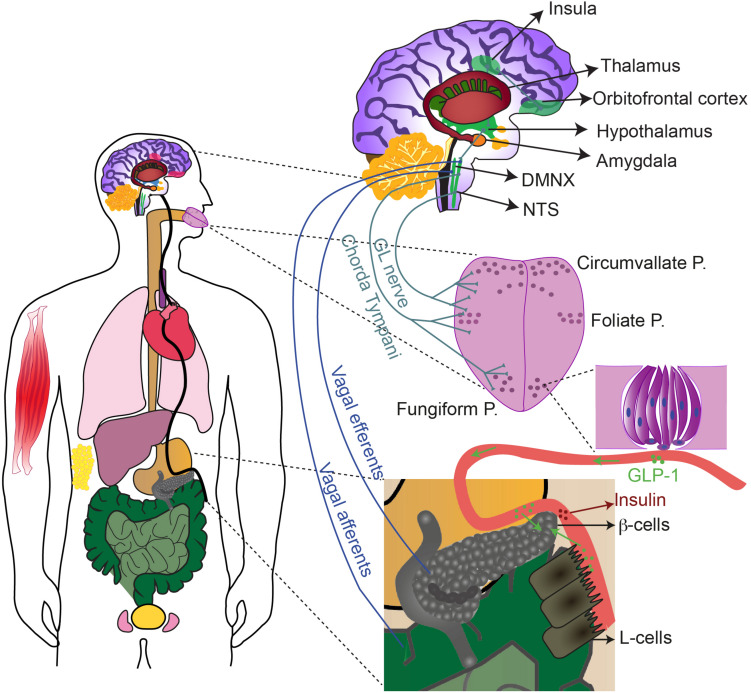
The sweet taste receptor contributes to gustation and extraoral sugar-sensing. T1R2/T1R3, and possibly T1R3 homomers, are expressed with their downstream signaling molecules in multiple extraoral tissues (in color), including: oesophagus, stomach, liver, pancreas, intestine, bladder, testis, skeletal muscle, respiratory tract and adipose tissue. Activation of sweet-sensitive taste bud cells leads to purinergic stimulation of the chorda tympani and the glossopharyngeal (GL) nerves that send information, upon relay in the NTS and the thalamus, up to the insula. The insula communicates with several other brain regions (some are depicted) to regulate reward, motivation and energy homeostasis. Taste bud cells release also GLP-1 that can activate the afferent fibers as well. Sweet stimulation of taste bud cells thereby generates CPIR, possibly via NTS-DMNX communication and consequent activation of efferent vagal fibers. GLP-1 released by taste bud cells in the circulation, may also reach the pancreas, the intestine and the brain, exerting there paracrine effects.

The alternative pathway may use not only a distinct intracellular signaling, but also a different neurotransmitter. A likely candidate for signal transmission from taste bud cells to gustatory nerves is glucagon-like peptide-1 (GLP-1) (Figures 1, 2). Indeed, GLP-1 and its synthetizing enzyme convertase, were detected in a subset of type II and type III cells in mouse circumvallate papillae (Feng et al., 2008; Shin et al., 2010; Kokrashvili et al., 2014) (for review von Molitor et al., 2020c), while the related receptor (GLP-1R) was found in intragemmal nerve fibers (Shin et al., 2008; Takai et al., 2015). GLP-1 released by taste bud cells is selectively sweet- and lipid-dependent and it potentiates sweet taste mediated attraction (Martin et al., 2009; Martin et al., 2012). Besides this, taste bud cells may contribute to systemic GLP-1, releasing it in the blood stream (Figure 2). In the body, GLP-1 is mainly released from enteroendocrine L-cells upon glucose uptake, via a mechanism involving SGLT1-mediated depolarization and activation of voltage-dependent calcium channels (VDCCs) (Best et al., 2010; Brubaker, 2017). Generally, GLP-1 controls fasting plasma glucagon, influences motoric mechanisms of gastric emptying, inhibits short-term food intake and potentiates pancreatic insulin release (Meier and Nauck, 2005; Schirra and Göke, 2005). In particular, GLP-1 induces the “cephalic phase insulin release” (CPIR). This is an innate response to sweet food ingestion that occurs prior its absorption, through which pancreatic insulin release occurs before blood glucose level rises (Louis-Sylvestre, 1976; Just et al., 2008). CPIR is important to prepare the body for ingestion, digestion, and storage of carbohydrate (Ahrén and Holst, 2001; Smeets et al., 2010). In healthy humans and rodents, CPIR is induced by oral exposure to sweet substances, but not to umami, salty or bitter compounds (Tonosaki et al., 2007; Just et al., 2008; Dušková et al., 2013). However, further studies are required to reveal the mechanism of GLP-1 release from sweet-sensitive taste bud cells and how this may induce CPIR (for review von Molitor et al., 2020c). Nonetheless, the alternative sweet-sensitive pathway could be a new interesting drug target (Laffitte et al., 2014; ZhuGe et al., 2020). If the idea holds true that taste bud cells mediate CPIR via oral secretion of GLP-1 (Chambers et al., 2017; Svendsen et al., 2018), controlling GLP-1 signaling in the tongue may help to control glycemia or even to treat diabetes (von Molitor et al., 2020c).

## Caloric Sugars and Non-Caloric Sweeteners May Utilize Distinct Pathways

Before the discovery of the sweet taste receptor, taste bud cells responses to caloric and non-caloric sweeteners were proposed to be mediated by two different pathways. In rat taste buds, caloric sugars were shown to augment cAMP concentrations, while non-caloric sweeteners mainly raised intracellular IP3 concentration (Striem et al., 1991; Bernhardt et al., 1996). Moreover, with Ca^2+^ imaging experiments, it was shown that extracellular Ca^2+^ is required only for nutritive sugar-mediated responses (Bernhardt et al., 1996). Thus, the response to sugars seems to involve the cAMP/PKA pathway and to depend on Ca^2+^ influx, while non-caloric sweeteners probably induce IP3-mediated Ca^2+^ release from the stores (Bernhardt et al., 1996). However, it remains elusive, whether such a strict separation holds true, since saccharin was reported to activate both pathways: at low concentrations the cAMP/PKA-signaling, and at higher concentrations the IP3-pathway (Nakashima and Ninomiya, 1999). Presumably, saccharin can switch its activity from sweet to bitter agonist in a concentration-dependent manner (Kuhn, 2004; Galindo-Cuspinera, 2006; Behrens, 2017). Since mice deficient for PLCβ2 (Zhang et al., 2003; Dotson et al., 2005) and TRPM5 (Zhang et al., 2003; Talavera et al., 2005; Damak et al., 2006) were shown to be insensitive to non-caloric sweeteners and to have largely reduced responses to natural sugars, PLCβ2 and TRPM5 may be crucial signaling molecules for perception of both caloric and non-caloric sweet tastants (for review von Molitor et al., 2020c). Furthermore, in rat taste buds the very same cells responded to sucrose and artifical sweeteners (Bernhardt et al., 1996; Lindemann, 1996). This can be interpreted as either both types of sweet stimuli activate the same pathway, or that two different pathways coexist in the same cell and interact. For example, GPCRs can mediate different signaling pathways via either, the α or βγ subunit (Zhu et al., 1994; Zhu and Birnbaumer, 1996). In support of the two-pathways hypothesis, non-caloric sweeteners mediated responses differ from those elicited by natural sugars as they have a higher potency, a delayed on- and off-set, and a lower sweetness intensity (DuBois et al., 1991; DuBois, 2016; Wee et al., 2018). Therefore, they are ranked less sweet than sugars by humans (Antenucci and Hayes, 2015) and mice (Smith and Sclafani, 2002). Stimulation of taste buds with sugars and non-caloric sweeteners also evokes different physiological responses. Natural sugars were able to induce CPIR in humans, as described in most studies (Goldfine et al., 1969; Berthoud et al., 1980; Yamazaki and Sakaguchi, 1986; Tonosaki et al., 2007; Just et al., 2008; Shinozaki et al., 2008; Dušková et al., 2013; Dhillon et al., 2017), but for non-caloric sweeteners this is still controversial (reviewed Han et al., 2019; von Molitor et al., 2020c). Along the same line, there is clear evidence that GLP-1 is released by taste bud cells upon nutritive sugar consumption, but a corresponding efficacy of non-caloric sweeteners is debated (for review Renwick and Molinary, 2010; von Molitor et al., 2020c). Furthermore, upon oral perception, both caloric sugars and non-caloric sweeteners activated the gustatory cortex, but their responses differed in terms of intensity and activated regions (Frank et al., 2008; Chambers et al., 2009). Finally, while natural sugars activated brain reward areas, such as the dopaminergic midbrain area (Frank et al., 2008) and the striatum (Chambers et al., 2009), non-caloric sweeteners failed to do so (for review Han et al., 2019). Considering the broad consumption of non-caloric sweeteners, it is crucial to find an answer to these many open questions to better interpretate and predict their physiological effect.

## Ca^2+^ Pathways in Taste Receptor Cells

Sweet taste responses rely on intracellular Ca^2+^ signals, that are translated into afferent-fiber activity in order to send the information to upper brain centers. In this context, type II cells released Ca^2+^ from the stores via PLC/IP3-mediated signaling upon saccharin stimulation (Bernhardt et al., 1996; Rebello et al., 2013). Consistent with this observation, neither VDCCs gene expression nor depolarization-induced Ca^2+^ signals were observed in type II cells (Clapp et al., 2006; DeFazio et al., 2006). However, others reported voltage-dependent Ca^2+^ influx in a subpopulation of bitter-responding cells, that may feature both type II and type III cells (Hacker and Medler, 2008) (for review Medler, 2015). Consistenly, the presence of “broadly responsive” taste bud cells has been recently reported, that are positive for the type III cell marker SNAP25, and respond to KCl-mediated depolarization with Ca^2+^ influx. Further, they responded to bitter, umami and/or sweet stimuli with Ca^2+^ signals mediated, in this case, by a different PLC: the PLCβ3. Thus, these “broadly responsive” cells feature both voltage-dependent Ca^2+^ influx and intracellular Ca^2+^ release, even if it remained elusive, wether they express VDCCs, T2R and T1R. In all papillae types, such cells represented about 20–30% of all taste bud cells and about 50% of type III cells. Furthermore, since PLCβ3-knockout mice have a reduced licking behavior to bitter, sweet and umami stimuli, this cell population crucially contributes to taste perception (Banik et al., 2018; Dutta Banik et al., 2020).

Further potential Ca^2+^ mobilization routes include: cyclic nucleotide gated (CNG) channels, store-operated Ca^2+^ channels (SOCs), vanilloid-receptor-1 (TRPV1), and Calcium Sensing Receptor (CaSR) (Figure 1 and Table 5).

**TABLE 5:** Possible Ca^2+^ signaling pathways in type II taste bud cells.

*Molecule*	*Activation*	*Effect*	*Source*
*VDCC*	depolarization	Ca^2+^ influx	Béhé et al., 1990; Medler et al., 2003; Hacker and Medler, 2008
*ORAI/STIM*	Ca^2+^ release from the store	Ca^2+^ influx	Ogura et al., 2002; Matsumura et al., 2009; Gilbertson and Khan, 2014; Ozdener et al., 2014
*TRMP5/TRPM4*	depolarization and Ca^2+^	Na^+^ influx and depolarization	Pérez et al., 2002; Prawitt et al., 2003; Talavera et al., 2005; Kaske et al., 2007
*CNG*	cAMP/cGMP	Na^+^ influx and depolarization	Kolesnikov and Margolskee, 1995; Misaka et al., 1997
*Ryanodine Receptor*	Ca^2+^, L-type VDCC	Ca^2+^ release from the store	Rebello and Medler, 2010; Rebello et al., 2013
*IP3R*	IP3	Ca^2+^ release from the store	Clapp et al., 2001; Hacker and Medler, 2008
*TRPV1*	capsaicine, temperature, H^+^	Ca^2+^, Na^+^, K^+^, Mg^2+^ influx	Lyall et al., 2004; Hacker and Medler, 2008; Laskowski and Medler, 2009
*CaSR*	glutation, Ca^2+^	Ca^2+^ release from the store	Maruyama et al., 2012; Medina et al., 2016

*Expression and functionality were assessed with RT-PCR (Prawitt et al., 2003; Hacker and Medler, 2008; Laskowski and Medler, 2009; Matsumura et al., 2009; Rebello and Medler, 2010; Maruyama et al., 2012) immunohistochemistry (Misaka et al., 1997; Clapp et al., 2001; Medler et al., 2003; Kaske et al., 2007; Hacker and Medler, 2008; Matsumura et al., 2009; Rebello and Medler, 2010; Maruyama et al., 2012; Rebello et al., 2013; Medina et al., 2016), in situ hybridization (Pérez et al., 2002), live-microscopy imaging (Ogura et al., 2002; Hacker and Medler, 2008; Laskowski and Medler, 2009; Rebello and Medler, 2010; Maruyama et al., 2012; Rebello et al., 2013; Ozdener et al., 2014; Medina et al., 2016), and electrophysiology (Béhé et al., 1990; Kolesnikov and Margolskee, 1995; Misaka et al., 1997; Medler et al., 2003; Prawitt et al., 2003; Lyall et al., 2004; Talavera et al., 2005).*

First, CNG channels are pivotal in decoding visual and olfactory sensations (Bradley et al., 2005). In frog taste bud cells, electrophysiological experiments have shown the presence of a CNG conductance that was inhibited by cAMP/cGMP. This argued for a sequence of events, whereby tastant-induced transducin activation would trigger PDE, leading to cyclic nucleotide degradation, CNG channel activation, depolarization and Ca^2+^ influx (Kolesnikov and Margolskee, 1995). In 1997, Misaka et al. cloned another CNG from rat tongue epithelial tissue that was present specifically in taste buds of circumvallate papillae at the pore side, whose expression disappeared upon glossopharyngeal nerve denervation. In contrast to the channel described by Kolesnikov and Margolskee, this channel was shown, in a recombinant system, to be activated by cGMP and cAMP (Misaka et al., 1997). However, it remained elusive whether it is able to conduct Ca^2+^, if it is functional in the native tissue and if it is linked to the sweet taste transduction pathway.

Second, SOCs may provide another way for Ca^2+^ entry into taste bud cells. In general, they are responsible for capacitive Ca^2+^ entry upon depletion of the store. In mouse taste bud cells, SOCs are composed of the proteins orai-1 and orai-3, which are under the control of an endoplasmatic-Ca^2+^ depletion sensor, i.e., stromal interaction molecule-1 (STIM-1). STIM-1 was shown to mediate the perception of fatty acids by the induction of Ca^2+^ signals (Dramane et al., 2012; Abdoul-Azize et al., 2014). Orai-1 and orai-3 are expressed in CD36 positive cells (Dramane et al., 2012), with CD36 and GPR120 being considered as receptors for fatty acids. In mouse, both receptors are expressed in some type II and type III cells (Matsumura et al., 2009; Gilbertson and Khan, 2014). They mediate Ca^2+^ signals and the release of serotonin and GLP-1 (Ozdener et al., 2014). Since long-chain fatty acids reinforce attraction to sugars (Martin et al., 2012), there might be a cross talk between signaling pathways triggered by fatty acids and sweet tastants. Indeed, TRPM5 may be a key signaling component in both pathways, since TRPM5-knockout mice had not only a reduced sweet taste sensitivity, but also an abolished fat preference (Sclafani et al., 2007). Interestingly, store-operated Ca^2+^ entry is involved in responses to prolonged bitter stimulation, meaning that not only Ca^2+^ release but also Ca^2+^ influx may be important for transduction (Ogura et al., 2002). Probably, this mechanism has been often underestimated, since the majority of electrophysiological and functional imaging experiments used only brief taste stimuli applications. Still, it is not known if prolonged sweet stimuli also require store-operated Ca^2+^ influx.

Third, the vanilloid-receptor-1 (TRPV1) is a non-selective cation channel permeable to Na^+^, Ca^2+^, K^+^ and NH_4_^+^ which is modulated by diverse stimuli such as vanilline, temperature, voltage and capsaicine. It was proposed to be responsible for salt detection, since it mediates amiloride-insensitive responses of the chorda tympani nerve not only to Na^+^, but also to Ca^2+^, K^+^ and NH_4_^+^ (Lyall et al., 2004). It is responsible for constitutive Ca^2+^ entry, which is then regulated by mitochondrial Ca^2+^ buffering and membrane Ca^2+^ extrusion via the Na^+^-Ca^2+^ exchanger (NCX) (Hacker and Medler, 2008; Laskowski and Medler, 2009). The concerted action of these players may contribute to the regulation of intracellular Ca^2+^ homeostasis in taste bud cells. In fact, both pharmacological alteration of the mitochondrial potential (Hacker and Medler, 2008) and blocking of NCX, induced Ca^2+^ signals in taste bud cells (Laskowski and Medler, 2009). Also, mitochondria were shown to differentially contribute to Ca^2+^ buffering in type II and type III cells (Hacker and Medler, 2008; Medler, 2015), and metabolic stimuli may affect intracellular Ca^2+^ homeostasis by interfering with mitochondrial activity: in type II cells, changes in mitochondrial potential induced by glucose metabolism may decrease mitochondrial Ca^2+^ buffering and affect intracellular Ca^2+^ transients (Hacker and Medler, 2008; Medler, 2015).

Fourth, an additional taste, called *kokumi*, makes use of the CaSR to induce specialized Ca^2+^ signals (Maruyama et al., 2012; Medina et al., 2016). CaSR is a typical GPCR which plays a central role in mammalian Ca^2+^ homeostasis (Chattopadhyay et al., 1997). It is present in both type II and type III cells, but it is not coexpressed with T1R3 (Bystrova et al., 2010; Maruyama et al., 2012). In rodents, most CaSR-positive cells were found in circumvallate and foliate papillae (San Gabriel et al., 2009). CaSR is activated by glutathione and cations such as Ca^2+^, Mg^2+^ and Gd^3+^. Although these CaSR agonists alone have no flavor, they enhanced the intensity of sweet or umami sensation. When recombinant CaSR was expressed in HEK cells, glucose and sucrose were able to elicit Ca^2+^ transients in the presence of extracellular Ca^2+^, suggesting that CaSR can be allosterically modulated by sugars to mobilize Ca^2+^ via a downstream pathway (Medina et al., 2016). Indeed, in mouse taste cells, *kokumi* substances induced intracellular Ca^2+^ release via PLC (Maruyama et al., 2012). CaSR can also be activated by bitter compounds (Rogachevskaja et al., 2011) and may, therefore, exert an ubiquitous and still largely unknown modulatory effect on several taste modalities.

## Presence and Role of Sweet Taste Receptor in Extraoral Tissues

Apparently, T1R2/T1R3 is not only responsible for sweet taste detection in the oral cavity, since it is also expressed in several extra oral tissues (Figure 2), together with its downstream signaling molecules (Table 6 and Figure 2) (for review Yamamoto and Ishimaru, 2013; Laffitte et al., 2014). Most of these tissues are involved in carbohydrate metabolism and there, the sweet taste receptor is involved in nutrient sensing, monitoring changes in energy storage and triggering metabolic and behavioral responses to maintain the energy balance (Lee and Owyang, 2017). Thus, the wide expression of the sweet taste receptor highlights potential health risks that sweeteners pose, due to their multiple targets in the body (Laffitte et al., 2014). The following paragraphs briefly introduce the main findings on extraoral expression of sweet taste receptors and the knowledge on their function there.

**TABLE 6:** Gastro-intestinal expression of sweet taste signaling molecules.

*Organ*	*T1R2*	*T1R3*	*T2R*	*Gα_*gust*_*	*PLCβ2*	*TRPM5*	*GLUT/SGLT*	*K*_*ATP*_	*Species*	*Source*
*Oeso- phagus*	✓		✓		✓					
			low		low				Human	Young et al., 2009
*Stomach*	✓	✓	✓	✓	✓	✓	✓	✓	Mouse	Hass et al., 2007; Kaske et al., 2007; Bezençon et al., 2008; Hass et al., 2010; Janssen et al., 2011; Widmayer et al., 2011; Sakata et al., 2012
*Intestine*	✓	✓	✓	✓		✓				
				low					Human	Bezençon et al., 2007; Jang et al., 2007; Young et al., 2009
	✓	✓	✓	✓			✓		Mouse	Dyer et al., 2005; Margolskee et al., 2007; Janssen et al., 2011
							✓	✓	Rat	Kuhre et al., 2015
*Colon*		✓	✓	✓					Human	Taniguchi, 2004; Rozengurt et al., 2006
	✓	✓	✓							
			low						Mouse	Bezençon et al., 2008; Reimann et al., 2008
						✓				
						low			Rat	Jie et al., 2015
*Pancreas*		✓				✓	✓	✓	Human	Prawitt et al., 2003; Taniguchi, 2004
	✓	✓		✓		✓	✓	✓	Mouse	Prawitt et al., 2003; Nakagawa et al., 2009; Colsoul et al., 2010; Nakagawa et al., 2014
							✓	✓	Rat	Cook and Hales, 1984; Inagaki et al., 1995; Vos et al., 1995

*Methods included: PCR (Prawitt et al., 2003; Dyer et al., 2005; Rozengurt et al., 2006; Margolskee et al., 2007; Bezençon et al., 2008; Nakagawa et al., 2009; Young et al., 2009; Widmayer et al., 2011; Sakata et al., 2012), in situ hybridization (Hass et al., 2007; Margolskee et al., 2007), immunohistochemistry (Taniguchi, 2004; Rozengurt et al., 2006; Hass et al., 2007; Jang et al., 2007; Kaske et al., 2007; Margolskee et al., 2007; Bezençon et al., 2008; Colsoul et al., 2010; Janssen et al., 2011; Widmayer et al., 2011), biochemical measurement (Kuhre et al., 2015), or transcriptome analysis (Jie et al., 2015).*

### Sweet Taste Receptors in the Gastrointestinal Tract

In the esophagus, mainly T1R3-homodimers are present (Young et al., 2009), while in the mouse stomach, T1R3 is expressed at higher levels than T1R2, supporting the hypothesis that homomeric and dimeric receptors may be both present (Hass et al., 2010). Gustducin expression in rat stomach, duodenum, and the pancreatic duct was already known in 1996 (Höfer et al., 1996). Later, Hass et al. identified a cluster of gustducin, PLCβ2 and TRPM5 expressing cells in the mouse stomach. Even if colocalization studies were not possible, they noticed that gustducin and TRPM5 positive cells were scattered, whereas PLCβ2 positive cells were restricted to a basolateral sub-compartment (Hass et al., 2007). PLCβ2 positive cells further expressed cytokeratin 18, a marker for brush cells (Hass et al., 2007). In a follow up study, they showed also T1R3 expression in this region (Hass et al., 2010). Thus, these brush cell clusters may have chemosensory function and support the gastric compartment to sense nutrients. This may not only initiate the appropriate gastric processes for digestion and regulate gastric emptying (Rozengurt, 2006; Kendig et al., 2014), but may also be relevant to transmit information to the hypothalamic nuclei governing food intake (Hass et al., 2010). Accordingly, gustducin and T1R3 are coexpressed with the hunger hormone ghrelin (Hass et al., 2010; Janssen et al., 2011). Concerning the sweet taste receptor independent pathway, mRNAs encoding GLUT1,4,5 and components of the K_*ATP*_ channel (Kir6.2 and SUR1), were also detected in the pool of gastric mucosal cells secreting ghrelin. However, activators or inhibitors of the K_*ATP*_ channel did not change ghrelin release, as shown with mouse ghrelinoma cells kept at low density (Sakata et al., 2012). Hence, both the canonical and the alternative pathway may play a role in a ghrelin releasing cells of the stomach.

RT-PCR results have shown that humans and mice similarly express T1R2, T1R3, gustducin, PLCB2 and TRMP5 in gastro-intestinal tissues, with the exception of T1R2 that was not detected in the stomach (Bezençon et al., 2007). In rodent intestinal cell lines, T1R2/T1R3 is coexpressed with α-gustducin (Dyer et al., 2005; Margolskee et al., 2007). Additionally, in humans, the sweet taste receptor is highly expressed in the jejunum and duodenum and to a lesser content in the ileum (Dyer et al., 2005; Young et al., 2009). Moreover, gustducin was detected in more than 90% of human L-cells, in less than 50% of K-cells, and in other cell types in the duodenum (Jang et al., 2007). Gustducin expression was most prominent in the mid-jejunum (Young et al., 2009), the place where carbohydrate-induced reflexes are likely to be initiated (Lin et al., 1989). Besides this, the expression of PLCβ2 (Young et al., 2009) and TRPM5 (Prawitt et al., 2003; Young et al., 2009) has been demonstrated in gastro-intestinal cells, where they might be involved in sugar sensing via canonical sweet signaling (Table 6; Dyer et al., 2005; Bezençon et al., 2007). While the release of ghrelin by T1R3-expressing brush and endocrine cells (Hass et al., 2010) seemed to be sweet taste receptor and gustducin independent in mice (Steensels et al., 2016), the canonical pathway may mediate the release of GLP-1. Indeed, intestinal cells, expressing T1R2 and T1R3, released GLP-1 (Dyer et al., 2005; Jang et al., 2007), which was reduced in T1R3-knockout mice and upon sweet taste receptor inhibition (Jang et al., 2007). Furthermore, T1R3 and gustducin were shown to regulate the expression of SGLT1 in enterocytes, since sugars and non-caloric sweeteners stimulated SGLT1 expression and glucose absorptive capacity in wild-type mice, but not in T1R3- or gustducin-knockout mice (Margolskee et al., 2007). More recently, glucose intake via GLUT2 was found to induce the release of GLP-1 in rat intestine via glucose metabolism and ATP-mediated closure of K_*ATP*_ (Kuhre et al., 2015). Thus, as in the taste buds, both sweet taste receptor dependent and independent pathways may coexist.

The expression of T1R3 and gustducin has been shown also in human enteroendocrine L-cells of the colon (Rozengurt et al., 2006). Curiously, in mouse colon cells, gustducin is coexpressed with TRPM5, but not with PLCβ2 nor T1R3 (Bezençon et al., 2008). Further, SGLT1 and K_*ATP*_ expression in rat colon cells is low (Reimann et al., 2008). As gustducin expression was found in L-cells, which secrete GLP-1 and peptide tyrosine tyrosine (PYY), sweet taste receptors there may be involved in energy homeostasis (Rozengurt et al., 2006; Rozengurt, 2006). Additionally, it was proposed that T1R3/T1R2, along with T2R, may play a role in the peristaltic reflex (Rozengurt, 2006; Kendig et al., 2014).

### Sweet Taste Receptors in the Pancreas

Another key player in glucose homeostasis is the pancreas, where increased blood glucose levels are sensed via GLUT2. Upon glucose transport into β-cells, oxidative phosphorylation occurs which increases intracellular ATP that in turn inactivates K^+^ channels to mediate cell depolarization. This mechanism links glycolysis to hormonal release, since subsequent VDCCs-mediated Ca^2+^ influx triggers insulin secretion (Yamamoto and Ishimaru, 2013; Laffitte et al., 2014). Additionally, the pancreas senses sweet compounds via T1R3 and T1R2, which are both coexpressed with gustducin, as shown in MIN6 cells and mouse β-cells (Nakagawa et al., 2009; Medina et al., 2014). However, low mRNA levels of T1R2 were detected, suggesting that here T1R3-homodimers may be present and contribute to sweet taste receptor function (Young et al., 2009; Medina et al., 2014). T1R3 activation was shown to increase ATP production by promoting mitochondrial metabolism (Nakagawa et al., 2014; Kojima et al., 2015). Stimulation of β-cells with fructose, sucralose or non-caloric sweeteners led to increased insulin blood levels (Nakagawa et al., 2009), an effect blocked by knocking out T1R3 or inhibiting it with gurmarin, suggesting T1R3 functionality in β-cells (Geraedts et al., 2012; Nakagawa et al., 2013; Medina et al., 2014). In addition, TRMP5-knockout mice showed diminished glucose-mediated insulin secretion (Colsoul et al., 2010). Thus, in the pancreas, the canonical sweet taste pathway may function in synergy with the GLUT-mediated pathway.

### Sweet Taste Receptors in Other Tissues

Sweet taste receptor expression has been additionally documented in multiple other tissues not directly involved in glucose homeostasis, such as respiratory tract (Lee and Cohen, 2014; Workman et al., 2015), liver (Taniguchi, 2004), testes (Gong et al., 2016), heart (Wauson et al., 2012), bladder (Elliott et al., 2011), skeletal muscle (Kokabu et al., 2017), and adipose tissue (Masubuchi et al., 2013; Figure 2). The role of sweet taste receptors in these tissues is reviewed elsewhere (Lee and Cohen, 2015).

Besides metabolic functions, taste receptors may play a role also in the innate immune response. In support of this, human solitary chemosensory cells (SCCs) expressed T2R and T1R receptors (Lee et al., 2014). SCCs are discrete, non-ciliated cells of the nasal respiratory epithelium (Yamamoto and Ishimaru, 2013; Maina et al., 2018). As shown in rodents, they express gustducin (Finger et al., 2003) and TRPM5 (Lin et al., 2008). During infection of the upper airways, gram-negative bacteria release bitter noxious substances, called acyl-homoserine lactones (AHLs), which are agonists of T2R38 (Lee et al., 2012). Accordingly, human neutrophils can identify AHLs via T2R38 (Maurer et al., 2015) and additional T2R members (Yan et al., 2017). T2R stimulation then leads to PLCβ2 activation and increased intracellular Ca^2+^ which spreads to neighboring ciliated cells via gap junctions to induce secretion of anti-microbial peptides for killing pathogenic microbes (Finger et al., 2003; Lee et al., 2014) (for review see Maina et al., 2018; Triantafillou et al., 2018). Stimulation of the sweet taste receptor, expressed in the same cells, led to inhibition of this defense pathway (Lee et al., 2014). However, when bacteria metabolize glucose, its concentration in the airway mucus decreases and this interrupts the tonic activation of T1R2/T1R3, boosting the immune response (Maina et al., 2018). Thus, the combination of sweet taste receptor antagonists with bitter receptor agonists could be a new potential pharmacological approach to treatment chronic rhinosinusitis or airway infections (reviewed in Workman et al., 2015; Maina et al., 2018).

The ubiquitous expression of sweet taste receptors indicates that sweet compounds and other allosteric binding partners of T1R2/T1R3 (Kojima and Nakagawa, 2011) and T1R3-homomeric receptors (Young et al., 2009; Medina et al., 2014) may induce potential health risks via inappropriate metabolic effects, such as: stimulating the release of gut or pancreatic hormones, altering glucose absorption, or modulating immune responses (Geraedts et al., 2012; Laffitte et al., 2014). The other way around, this may open new theraputical prospectives for the treatment of obesity related metabolic disfunctions (Laffitte et al., 2014; Workman et al., 2015; Maina et al., 2018).

## How Sugars Affect the Brain

The ability to detect sugars is crucial in human nutrition as it orients food choice and energy intake. Sugars not only provide the energy necessary for metabolism, but also guide the behavior. Our preference for sugars is innate (Steiner et al., 2001), but affective responses to flavors are acquired based on experience (Araujo et al., 2020), allowing the organism to learn which food is rich in energy. Further, sugar preference mediates attraction and reward mechanisms (for review Gutierrez et al., 2020). Thus, both nutritional and sensory properties regulate food intake.

Sugar preference and intake are controlled at least on three levels: gustation, gut-brain axis and brain-glucose sensing. The brain can sense glucose either directly or indirectly via oro- and visceral-sensation. Sensory, hedonic and metabolic values are encoded by separate brain circuitries working in parallel (Araujo et al., 2012) (for review Han et al., 2019; Gutierrez et al., 2020). Notably, preference for sugars does not seem to depend on its caloric content (Wright et al., 2011; Zukerman et al., 2013) nor on sweet taste receptors (Araujo et al., 2008; Ren et al., 2010; Oliveira-Maia et al., 2012; Tan et al., 2020), and even if food palatability affects what we eat, it does not influence how much we eat (Araujo, 2016). Rather, post-oral mechanisms are critical in controlling sugar intake and establishing long-term preference (Araujo, 2011). Therefore, different factors and pathways appear to regulate our preference for sugars on the one hand, and the amount of sugar consumption on the other hand.

### Gustatory Representation

Consciously, sweetness can be perceived only upon activation of the sweet taste receptor in the oral cavity. Therefore, type II cells communicate with afferent gustatory fibers; these send the information, via several relay stations, up to the cortex (Figure 2; Ohla et al., 2019). How the taste quality is conveyed to the brain, is still a matter of debate. Yet, it is widely accepted that taste bud cells are hardwired to a defined behavior, i.e., not the identity of the taste receptor but of the perceiving cells determine the behavioral response. For example, when an opiate (Zhao et al., 2003) or a bitter-taste receptor (Mueller et al., 2005) was expressed in type II sweet-sensitive cells of mice, these transgenic animals were attracted by tasteless synthetic opiates or by bitter tastants, respectively. Thus, it was thought that sweet-responding taste bud cells respond only to this taste modality, however this view has been recently challenged by the discovery of broadly responding taste cells (Banik et al., 2018; Dutta Banik et al., 2020).

Afferent neurons may respond either to only one (best stimulus) or to multiple qualities (broadly tuned), and their tuning may vary according the stimulus concentration (Barretto et al., 2015; Wu et al., 2015). The cell bodies of the afferent neurons are located either in the geniculate, petrosal or nodose ganglia, projecting to the rostral portion of the solitary tract nucleus (rNTS) (Corson and Erisir, 2013). In human, the rNTS secondary neurons send their axons directly to the parvocellular portion of the Vetroposteromedial nucleus of the thalamus (VPMpc), while in rodents they make a first relay in the parabrachial nucleus (Samuelsen et al., 2013). From the thalamus, the information is conveyed to the primary gustatory cortex, called insula (IC), that further projects to the orbitofrontal cortex (for review Small, 2012; Ohla et al., 2019; Figure 2).

Electrophysiological and live-imaging experiments in rodents have shown that along the neural axis, both specialized and generalized neurons encode the sweet taste (Spector and Travers, 2005; Ohla et al., 2019). Moving toward higher brain centers, a growing percentage of neurons responds to multiple stimuli (broadly tuned). Here, combinatorial and temporal coding are crucial for taste decoding, thus the neuronal ensembles and their firing frequency pattern decode important gustatory information (Spector and Travers, 2005; Stapleton et al., 2007; Ohla et al., 2019). Accordingly, sweet taste intensity is decoded by neuronal firing frequency in the insular cortex and in the orbitofrontal cortex (Fonseca et al., 2018). In human, a gustatopic map has been recognized in the insula, where discrete regions were activated by oral exposure to a defined taste modality, and even the concentration intensity was represented by a spatial gradient (Prinster et al., 2017; Canna et al., 2019; Chikazoe et al., 2019; Porcu et al., 2020). On the contrary, other data support rather a distributed pattern of activity (Ohla et al., 2019; Porcu et al., 2020). Accordingly, no insular region revealed a consistent preference for a specific taste quality (Avery et al., 2020) and taste representation was not only highly variable across subjects (Schoenfeld et al., 2004; Avery et al., 2020), but also within subjects on different days (Avery et al., 2020). It is controversially discussed also for rodents whether the different taste modalities are encoded by topographically distinct cortical fields (Chen et al., 2011; Fletcher et al., 2017; for review Ohla et al., 2019). Optogenetic stimulation of the sweet responsive insular region induced increased licking of water in mice, suggesting that they perceived it as a sweet solution (Peng et al., 2015). Thus, the internal representation in the insula may underlie innate sweet preference. The insula not only encodes the chemical identity, but also the palatability of tastants (Araujo et al., 2006) and communicates with higher and lower-order neural relays, such as the striatum (Small et al., 2003; Oliveira-Maia et al., 2012) and the orbitofrontal cortex (Haase et al., 2008). Thus, as shown by electrophysiolgical recordings in mice, palatability is encoded by a widespread network including multiple brain regions (Parabrachial Nucleus, VPMpc, Basolateral Amygdala, Nucleus Accumbens Shell and lateral hypothalamic area) (for review Gutierrez et al., 2020). The coordinated action of these neural pathways motivates sugar intake. Furthermore, the insula preferentially interacts with the hypothalamus when the stimulus is nutritive (Rudenga et al., 2010). While it is still discussed whether the sweet taste itself can stimulate hunger (for review Low et al., 2014), sugars can induce attraction even in the absence of sweet taste receptor-mediated oral sensation, as shown with different ageusic transgenic mice (Nelson et al., 2001; Araujo et al., 2008; Sclafani et al., 2014). Oral sweet-sensing influences the initial food acceptance, but it does not determine the daily caloric intake (Glendinning et al., 2010; Ren et al., 2010). Other senses also influence sweet perception and hedonic value, via multisensory integration in the NST (Travers and Norgren, 1995), in the insula and in the orbitofrontal cortex (Rolls and Baylis, 1994) (for review Small, 2012). Sweet perception is additionally influenced by expectation (Veldhuizen et al., 2007), emotions (Noel and Dando, 2015), and metabolic state (Zhang et al., 2018).

### Gut-Brain Axis

To feel pleasure and develop attraction to sugars, taste recognition needs to be integrated with energy-value sensing. Preference for a certain flavor develops only when its taste is paired to post-ingestive reward signals (for review Kim et al., 2018; Gutierrez et al., 2020). Accordingly, mice develop a preference for sugars over non-caloric sweeteners within 48 h, when solutions, perceived equivalenty sweet, were provided (Tan et al., 2020). The mechanism involved is also responsible for sugar craving (Volkow et al., 2011). Sensing of the metabolic value occurs in the gastro-intestinal tract and/or the portal vein, both sending signals via vagal afferent fibers to the NTS and from here to upper brain centers. Specifically, in mice, proenkephaline-positive neurons of the caudal nucleus of the solitary tract (cNTS) were strongly activated by ingestion or intragastric application of sugars, but not of non-caloric sweeteners. This was mediated by vagal sensory neurons of the nodose ganglia receiving input from the duodenum and synapsing to cNTS excitatory neurons (Tan et al., 2020). Silencing these vagal afferents or the proenkephaline-positive neurons prevented the development of sugar preference, but left innate sweet attraction intact. Thus, innate sweet attraction and learned sugar preferences use different neural circuitries. Substrates of SGLT1, such as non-metabolizable 3-OMG and galactose, were also able to activate vagal sensory neurons in the intestine. The lack of responses to non-caloric sweeteners, fructose and mannose further supported the involvement of SGLT1, since they are no substrates of SGLT1 (Tan et al., 2020). Consistently, in SGLT1-deficient mice, flavor conditioning to glucose was impaired, while natural preference was not affected (Sclafani et al., 2016). Tan et al. further provided evidence that this neural circuitry in mice is involved in the development of novel preference: when a stimulus was paired with chemo-genetic activation of cNTS proenkephaline-neurons, it became the preferred stimulus, thus less sweet solutions were preferred over sweeter ones, while silencing their synaptic activity prevented the development of preference (Tan et al., 2020). However, non-caloric sweeteners failed to activate the post-oral reward circuitry and to induce incretin hormone release, that mediates CPIR and satiety signals (for review Pepino and Bourne, 2011; Low et al., 2014). The uncoupling of gustatory signal and metabolic value, that occurs with non-caloric sweeteners, seems to alter reward and satiation responses also in human (for review Han et al., 2019). This may explain some adverse effects of non-caloric sweeteners and why they are not so effective in reducing weight (Swithers and Davidson, 2008; Lohner et al., 2017). In summary, visceral signals regulate feeding independently of the sweet taste receptor and its downstream signaling (Araujo et al., 2008; Ren et al., 2010; Oliveira-Maia et al., 2012; Tan et al., 2020). Thus, although post-oral sugar sensing does not convey taste perception, it is important to develop preference and it activates also neurons in the cortical region responsible for gustation. Indeed, activation of the dorsal insula is required to develop sugar preference in ageusic mice (TRPM5-knockout) with a conditioning protocol (Oliveira-Maia et al., 2012). Recent evidence suggests that sweetness and nutritional signals engage distinct brain networks, to motivate ingestion, both in mice and in humans (Tellez et al., 2016; Thanarajah et al., 2019).

Indeed, a recent study in healthy subjects combined fMRI and PET imaging to show that orosensory and post-ingestive mechanisms underly two distinct peaks in dopamine and recruit segregated brain circutries. The first dopaminergic response involved the dorsal striatum, the mesolimbic system, the orosensory pathways and areas participating in reward value signaling, while the second, delayed response was visible in distinct regions, such as the amygdala and the caudate nucleus (Thanarajah et al., 2019). In mice, sweet tasting stimulated dopamine release in the ventral striatum, via projection of the ventral tegmental area, while nutritional visceral sensing induced dopamine release in the dorsal striatum from the Substantia Nigra pars compacta neurons (SNpc). Thus, mesolimbic and nigrostriatal pathways detected taste and food energy, respectively (Tellez et al., 2016). However, the level of dopamine released in both regions was dependent on glucose oxidation rates, and glucose induced higher dopamine release compared to isocaloric serine. Thus, carbohydrate-specific preference can develop independently of taste quality or caloric load. Rather, it is associated with the ability of the body to use carbohydrates as a fuel (Ren et al., 2010). Similar results were obtained in humans with fMRI imaging, showing that glucose metabolism was a critical signal for regulating NAc and hypothalamic responses to food cues, independently of flavor liking (Araujo et al., 2013). However, the exact pathway linking sweet visceral sensing to dopamine release in the brain still needs to be elucidated in humans.

Recently, a pathway connecting gut-chemosensation to brain reward-circuitries was proposed in mice. It involves vagal afferent fibers originating from the stomach and the duodenum, which project, via the right nodose ganglia, to the ventromedial area of the NTS. At the end, via further projections, the dopaminergic neurons of the SNpc are activated to release dopamine in the dorsal striatum (Han et al., 2018). However, it is unlikely that this pathway is activated by sugars, since subdiaphragmatic vagotomy did not alter the preference to glucose (Qu et al., 2019). Rather, the mesenteric portal system, which transports glucose from the proximal intestine to the liver, may be crucially involved in food preference acquisition. Mechanistically, this might involve glucose sensing via SGLT1 and GLUT2 followed by information transmission to upper neural stations (Berthoud, 2004; Zukerman et al., 2013; Sclafani et al., 2016; Zhang et al., 2019). Further, bypass surgery of obese people has brought evidence that the gut-brain axis regulates food reward and motivation to eat also in humans (for review Orellana et al., 2019). In Roux-en-Y gastric bypass (RYGB), a small pouch of the stomach is connected with a distal part of the small intestine. This surgery shows the higher efficacy in inducing large and permanent reductions in body weight. Notably, this is not only due to reduced volume and absorbtion of the ingested food, but also to neural and hormonal changes (Ochner et al., 2011; Goldstone et al., 2016; Kim et al., 2018; Tsouristakis et al., 2019). Presumably, both neural and hormonal mechanisms are responsible for the enhanced intake of healthy food as well as for the selective reduction of high-caloric food preference and of hunger, which are observed in RYGB patients (Nance et al., 2020). It appears, that such behavioral changes have a neural correlate. Indeed, RYGB seems to restore the balance in the dopaminergic reward systems, which is altered by overeating. In obese people, the availability of striatal dopaminergic D2R is reduced (Wang et al., 2001), while several prefrontal cortex regions are overactivated upon meal consumption or exposure to food cues (Tomasi and Volkow, 2013) (for review Volkow et al., 2011). Conversely, RYGB reduces prefrontal cortex activation and may restore striatal D2R availability (Hamilton et al., 2018). In summary, postingestive sugar sensing is important for body homeostasis as well as sweet gustation, but the exact mechanisms underlying post-oral learned sugar preference still need to be elucidated.

### Brain Glucose Sensing

Glucose is the preferred brain fuel. Substituting it with lactate, the major metabolic alternative for neurons, specifically impaired neuronal network activities that require high energy expenditure, such as gamma- and theta-oscillations (Hollnagel et al., 2020). Thus, only glucose supports optimal information processing in awake animals. In addition to this, some brain areas have specialized neurons working as “glucose sensors”: their firing activity is indeed regulated by extracellular glucose levels. Possibly, these neurons are not activated by intracellular glucose metabolism, but rather by sensing extracellular glucose via the sweet taste receptor (Ren et al., 2009; Kohno, 2017). The genes of the sweet taste receptor subunits, *Tas1r2* and *Tas1r3*, and of the associated G-protein gustducin are active in several mammalian brain regions: most abundantly in the hypothalamus, but also in the hippocampus, the habenula, the cortex and the epithelial intraventricular cells of the choroid plexus (Ren et al., 2009).

The hypothalamus is a major regulator of energy balance, as it regulates food intake and metabolism (Kohno, 2017). Here, the first step in energy regulation, i.e., energy sensing, is executed by direct neuronal glucose sensing: some neurons respond to high glucose with excitation and some with inhibition (Fioramonti et al., 2007). It is still open, if this is directly linked to hunger induction, however, two hypothalamic neuronal populations might be particularly relevant in that context: AgRP (orexigenic neuropeptide Y/agouti-related peptide) and POMC (proopiomelanocortin) neurons located in the arcuate nuclei (ARC). Transition of AgRP neurons from active to inactive states was proportional to calories ingested, driving hunger and promoting food intake (Chen et al., 2016; Beutler et al., 2017). Via multiple downstream relay stations, AgRP neurons control also the neuronal activity in the insula, influencing food salience (Livneh et al., 2017). Conversely, POMC neuron activation led to suppression of appetite and food intake (Aponte et al., 2011). Several mechanisms have been described underlying these effects, some related to glucose metabolism and ATP production, and some independent of it, possibly mediated by the sweet taste receptor (for review see Kohno, 2017). Specifically, in the hypothalamus, T1R2 and T1R3 expression was regulated by the nutritional state: it was increased under food deprivation and decreased upon obesity (Ren et al., 2009). Mimicking this situation *in vitro*, exposure to a low glucose medium selectively promoted higher T1R2 expression in a mouse hypothalamic neuronal cell line, while hyperglycemic media reduced its expression, independently of glucose metabolism (Ren et al., 2009). The non-caloric sweetener sucralose regulated T1R2 expression as well, confirming a metabolism-independent pathway (Ren et al., 2009). Overall, this suggests that sweet taste receptor expression in hypothalamic neurons is under the control of ligand concentration and energy status. However, the regulation is bidirectional as activation of the sweet taste receptor in the ARC hypothalamic nuclei controls neuronal activity and, thus, food intake. In particular, mainly non-POMC leptine-responsive neurons in mice responded to high glucose and/or sucralose with Ca^2+^ increase that was mediated by the sweet taste receptor and L-type Ca^2+^ channels (Kohno et al., 2016). However, in 33% of these neurons the response was not blocked by gurmarine, which specifically interacts with gustducin to block sweet signal transduction, therefore additional mechanisms may be involved (Kohno et al., 2016). Further studies are required to unravel the neuronal types in the ARC that respond to glucose and their physiological role in controlling hunger and satiety.

In summary, sugars can motivate their consumption independently of their sweet taste. Paring signals arising from the tongue and from the gastrointestinal systems is a way to develop and reinforce preference for sugars. Reinforcement occurs when taste is coupled to nutritional value sensing, assuring that absorption, metabolism and energy production follow gustation.

## The Journey of Unraveling Sweet Taste

**FIGURE 3:**
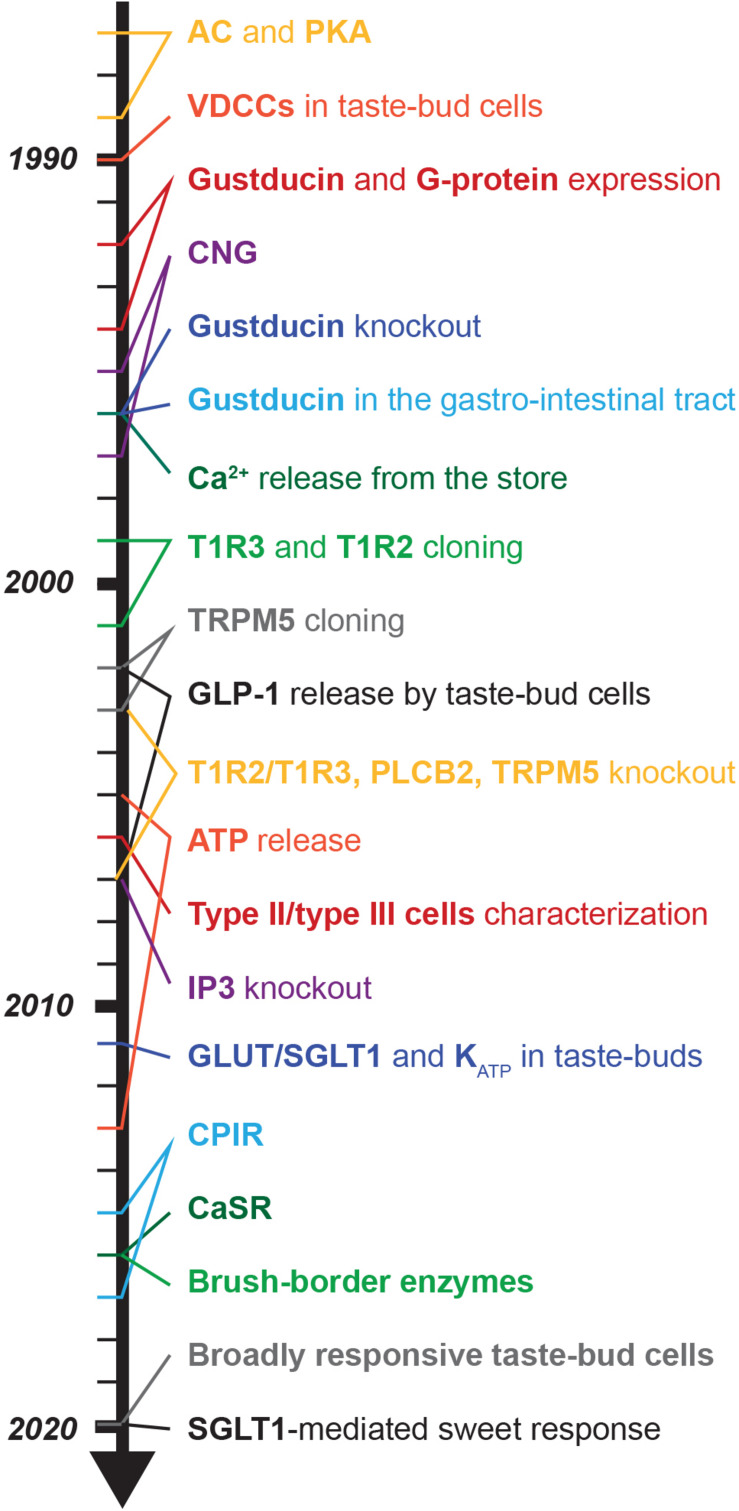
Time-line of the most important findings in sweet taste signaling. The years of the most important publications are marked by lines. The scheme refers to findings related to sweet signaling transduction and focus mainly on the taste bud cells.

The field of sweet gustation research can be divided in two eras: before and after the discovery of the sweet taste receptor (Figure 3). The cloning of the sweet taste receptor, which occurred at the beginning of this millenium, was the result of a longstanding research and opened new perspectives, new methods and new approaches (Temussi, 2006; von Molitor et al., 2020c).

However, investigation of sweet taste signal transduction has started already in the 70s (Table 6), long before the sweet taste receptor was identified. These studies revealed the involvement of the cAMP/PKA pathway in sweet transduction in different species (Kurihara and Koyama, 1972; Avenet and Lindemann, 1987; Tonosaki and Funakoshi, 1988). In the 90s, gustducin was discovered (McLaughlin et al., 1992; Takami et al., 1994) and it took further ∼10 years of intensive research to finally unravel the identity of the sweet taste receptor (Bachmanov et al., 2001; Kitagawa et al., 2001; Max et al., 2001; Montmayeur et al., 2001; Nelson et al., 2001; Li et al., 2002). This was followed by a boost of research which progressively uncovered the components of the PLCβ2/IP3 downstream-pathway. This was made possible by the parallel discoveries on bitter-mediated signaling (Table 2). Thus, many assumptions true for bitter transduction were transferred to sweet signaling, even if there were only weak or even contradictory evidences. At some point, it became a kind of common sense that sweet, bitter and umami receptors share a similar intracellular signaling mechanism, called “canonical pathway.” Since bitter, sweet and umami receptors were found to be mainly expressed in different subgroups of type II cells, it was proposed that the specificity of the response is assured by the type of taste bud cell activated (Finger, 2005). Furthermore, sweet-responsive taste bud cells were shown to be hard-wired via a specific neural connection to a defined behavioral response (Zhao et al., 2003; Mueller et al., 2005). Thus, the first 10 years from the discovery of the sweet taste receptor were mainly dedicated to the characterization of the cell types (Finger, 2005; Clapp et al., 2006; DeFazio et al., 2006), the downstream molecules (Table 2) and the mechanism of taste bud cells communication (Finger et al., 2005; Huang et al., 2007; Taruno et al., 2013) with afferent fibers and neighboring cells. This reinforced the concept and the importance of the “canonical pathway,” but it also marginalized the involvement of the cAMP/PKA-signaling and alternative viewpoints in sugar-mediated responses. A simplified interpretation of sweet taste coding in the taste buds was put forward: one-signaling mechanism and one-cell type (Zhao et al., 2003; Mueller et al., 2005).

However, it became increasingly clear that the picture was much more complicated. Knockout mice models for T1R2/T1R3 (Zhao et al., 2003) and its downstream molecules such as gustducin, PLCβ2 (Zhang et al., 2003), IP3R (Hisatsune et al., 2007), and TRMP5 (Zhang et al., 2003), verified their functional roles in sweet taste transduction, but residual responses to caloric sugars and not to non-caloric sweeteners (Damak et al., 2003) called for alternative mechanisms responsible for oral-mediated sweet gustation (Glendinning et al., 2015, 2017; Yasumatsu et al., 2020) (for review von Molitor et al., 2020c). In analogy to the mechanisms of glucose-sensing in extraoral tissue, some possible players, such as SGLT1, GLUT2, and K_*ATP*_ channels, have raised interest starting from the 2011 (Merigo et al., 2011; Yee et al., 2011), since they are expressed in taste bud cells, however their function is still debated (for review von Molitor et al., 2020c). Nonetheless, a physiological role was proposed for this alternative pathway, as it could be important to recognize the caloric value of food already in the mouth, and to drive CPIR (Glendinning et al., 2015, 2017). At the same time, sweet taste receptor expression was detected in many other organs (for review Yamamoto and Ishimaru, 2013; Laffitte et al., 2014). Since then, most publications have focused on studying taste transduction in extraoral tissues as they may offer new therapeutic possibilities. Accordingly, less projects still focus on fundamental sweet-signaling in taste bud cells, though there are still many open questions, even about the canonical signaling pathway. From the broad extraoral expression of sweet taste receptor, it became evident that the effects of sugars and non-caloric sweeteners are mediated not only by gustation, but also by sweet visceral-sensing (Araujo et al., 2008; Ren et al., 2010; Oliveira-Maia et al., 2012). Thus, for optimal energy homeostasis, glucose sensing in the tongue, intestine, pancreas and the brain need to be coordinated in order to drive the appropriate behavior. Recently, it was proposed that attraction to sugars is not only linked to conscious perception of sweetness, but also to visceral sugar-sensing, being especially relevant for the activation of reward circuitries and for learned sweet-preference (Kim et al., 2018; Han et al., 2019; Gutierrez et al., 2020). If this novel concept can be applied to human, it will challenge the whole interpretation of food-associated diseases, such as obesity and diabetes type II, and it will open new perspectives for their treatment (Neiers et al., 2016). We have also started to understand that non-caloric sweeteners, causing metabolic disregulation, increase the risk of these diseases (Renwick and Molinary, 2010; Pepino, 2015; Lohner et al., 2017). Consistently, extraoral sweet taste receptors are now in the focus of pharmaceutical industry (Sprous and Palmer, 2010). However, we should not stop investigating the sweet-signaling pathways in taste bud cells, since many questions are still open. Furthermore, discovering alternative sweet-sensitive taste mechanisms, their functional role and their ligands, may open the possibility to control energy homeostasis and our eating behavior already at the level of the mouth. In this context, it will be important to develop new and more physiological *in vitro* models to study sweet taste transduction in human.

## Author Contributions

EM and TC contributed to conceptualization, writing, and visualization. KR, MH, and RR contributed to writing – review and editing and contributed to supervision. RR contributed to project administration. MH and RR contributed to funding acquisition. All authors have read and agreed to the published version of the manuscript.

## Conflict of Interest

KR and MK were employed by the company BRAIN-Biotech. The remaining authors declare that the research was conducted in the absence of any commercial or financial relationships that could be construed as a potential conflict of interest.
